# Lisa Haushofer, *Wonder Foods: The Science and Commerce of Nutrition*, Oakland: University of California Press, 2023, ISBN: 9780520390409, 270 pp.

**DOI:** 10.1007/s10739-023-09756-2

**Published:** 2023-12-01

**Authors:** Joseph Bishop

**Affiliations:** Princeton University, Princeton, USA

Today’s popular connotations of early 20th century inventions commonly evoke x-rays, the Ford Model T, the radio, and perhaps the vacuum cleaner. But Lisa Haushofer has her sights trained on cornflakes and beef biscuits. In *Wonder Foods*, she traces how entrepreneurs pioneered food products designed to appeal to the public’s increasing reverence for science, and leveraged the commercial market’s growing control over food and nutrition science. Novel food technologies that arose between 1840 and 1940, particularly enchanted with economic reasoning, prompted the explosion of wonder foods—foods promising technical and nutritional solutions to politically fraught social issues.

Wonder foods were not merely feats of technological innovation and scientific creativity; they were products of Western industrialized countries’ commitment to capitalism, resource extraction, and white supremacy (p. 6). Haushofer explains how the modern diet became economized and uncovers its links to settler colonialism, race betterment programs, and neglected nutritional contributions of Indigenous people. She explores these themes in the United States and England, offering insightful transnational comparisons of each nation’s nutrition science and imperial ambition.

Readers of the *Journal of the History of Biology* may gravitate to Haushofer’s discussion of digestion and the products manufactured to facilitate the metabolism of food. Her work contributes to the history of the life sciences by considering central questions of scientific authority and its applications in commercial food, food processing, and the cultural force that food and diet have in US history. *Wonder Foods* is part of a current trend demonstrating that food is a serious historical subject and worth consideration from historians of science. In 2018, Haushofer co-authored an introductory essay for an issue in the *Journal of the History of Medicine and Allied Sciences* dedicated to recognizing the value of studying food as a historical object. Two years later, *Osiris*, an annual journal for historians of science, devoted an issue to food in history. Recent books overlapping in approach and subject matter are Benjamin Cohen’s *Pure Adulteration*, Anna Zeide’s *Canned*, and Nicholas Bauch’s *A Geography of Digestion*.

The commercial and imperial backdrop steered nutrition research toward isolating food’s constituent nutrients. Haushofer introduces Gail Borden, the inventor of condensed milk, and examines the impact of one of his lesser-known inventions: the meat biscuit. Borden claimed his meat biscuit condensed meat into a minimal space while maintaining its nutritional value, providing efficient and manageable food transportation. After patenting the meat biscuit, he marketed this “imperial technology” to the US military, preoccupied in the 1840s with Western expansion and conflicts with the Spanish and Native Americans (p. 43).

Unlike the American concern for increased mobility to enhance military capability into the West, British experts focused on waste reduction and increasing efficiency to maintain Britain’s imperial grip (p. 47). Experts sought to solve Britain’s food crisis by recasting waste products and discovering new food sources. They hoped science and technical means could alleviate the problem while evading the crisis’s fundamental causes of poverty, social inequality, and land privatization (p. 70).

The most robust section of the book examines Kellogg’s early-twentieth-century food product line as tools to provide the scientific and technological means for Kellogg’s aspiration for “race betterment,” a euthenics position that sought so-called racial improvements through environmental enhancements (p. 110–111). Kellogg’s first attempt at creating naturally predigested foods integrated the food practices of the Quechan people, the religious reforms of Presbyterian minister Sylvester Graham, and digestive physiological research. These foods incorporated the concepts of primitive and modern, and Kellogg claimed they restored digestion to full capacity. As predigested foods and digestive aids began to symbolize the weakening of the human constitution brought on by the easy living of modern civilization, Kellogg sought to stimulate the digestive system into action and avoid digestive aids that only reinforced the degeneration created by decadent modern society.

After receiving criticism from heredity-centric eugenicists that predigested foods exacerbated race degeneration by encouraging gluttony and laziness, Kellogg switched to foods that enhanced the digestive system’s natural functions (p. 111). Following the lineage of Grahamite health reformers, Kellogg created “peptogenic” food products with natural digestive capabilities (p. 145). This approach contrasted to the open admission by British companies, like Benger’s Food, of providing artificially digested food products. Kellogg sought to revert Americans’ health to a developmental stage between premodern culture and modern civilization— a move he called “a mild return to savagery” (p. 120).

Kellogg’s transition from his religious past to a modern focus on technological and scientific innovation with an entrepreneurial zest occurred as he invented his famous cereal flakes (p. 126). He constructed a philosophy that extolled consumption as an occasion to display discipline, self-control, and self-knowledge and painted consumerism as an opportunity for self-development. Of course, Kellogg provided an entire product line to help consumers in their self-development journeys via consumption (p. 146).

In the final section, Haushofer explains how, in the 1910s, yeast products diverged into two consumer trajectories that reflected a broader pattern of 20th-century wonder foods. The first path marketed yeast as a health food aimed at wealthy consumers in prosperous countries (p. 148). New consumer-focused ad techniques, such as the “yeast for health campaign” by the Fleischmann Company, largely shaped yeast as a particular health food serving customers for ailments that were not severe enough to warrant a doctor’s visit but irritating enough to be addressed. The second route shaped yeast into a cheap dietary supplement for poorer consumers in the Global South. The British Colonial Office and international health organizations considered a version of brewer’s yeast, called food yeast—a cheap, high protein, and vitamin-rich food—that could be distributed in the colonies to prevent “colonial malnutrition” (p. 149).

A particularly fascinating passage discusses the friendship between physiologist Ivan Pavlov and Kellogg. Pavlov once visited Kellogg and checked into Kellogg’s Battle Creek Sanitarium. The relationship between these two scientists cries out for further elaboration, especially considering that Pavlov also manufactured digestive enzymes from the stomachs of the dogs on which he experimented and sold the product as a lucrative digestion aid.

Haushofer excels in her coverage of Kellogg’s transition from a research physician into an entrepreneur, establishing a commercial product line of health foods and digestion aids. She also reveals Kellogg’s appropriation of Quechuan food preparation techniques, for which Kellogg did not give them credit. *Wonder Foods* achieves its purpose through a compelling narrative that explains how the modern diet became commodified, entangled with, and shaped by the priorities of maturing industrial capitalist markets and imperial expansion.

Haushofer demonstrates how the economic turn in food products connected health and productivity. Food culture espoused the accumulation of health, energy, and productivity from what we eat. Food had become “a commodity, a commercial opportunity, a means to engineer populations and environments and eat oneself to civilization and whiteness” (p. 185). *Wonder Foods* is well-written, clearly organized, and generously cited with reputable sources—an exemplary food history from the perspective of the history of science and medicine.

